# Granulomatous Mastitis With Erythema Nodosum During Pregnancy: A Case Report

**DOI:** 10.7759/cureus.24990

**Published:** 2022-05-14

**Authors:** Sayaka Mabuchi, Ryuichi Ohta, Keiko Egawa, Yoko Narai, Chiaki Sano

**Affiliations:** 1 Clinical Education and Research Center, Shimane Prefectural Central Hospital, Izumo, JPN; 2 Community Care, Unnan City Hospital, Unnan, Shimane, JPN; 3 Obstetrics and Gynecology, Shimane Prefectural Central Hospital, Izumo, JPN; 4 Community Medicine Management, Shimane University Faculty of Medicine, Izumo, JPN

**Keywords:** cortisol, prednisolone, drainage, pregnancy, erythema nodosum, granulomatous mastitis

## Abstract

Granulomatous mastitis is a rare benign disease that typically occurs in parous women. Some reports have described cases of erythema nodosum appearing following granulomatous mastitis, which is often treated with steroids. Here, we report a case of granulomatous mastitis with erythema nodosum successfully treated via drainage only, which may have been caused by the higher plasma cortisol levels observed during pregnancy. Although mastitis is rare during pregnancy, the current case suggests that granulomatous mastitis should be considered in pregnant women with treatment-resistant mastitis, especially in those with erythema nodosum and a history of birth. Furthermore, patients with granulomatous mastitis may not require prednisolone treatment during pregnancy, which may help in preventing steroid-associated conditions such as infections and gestational diabetes mellitus.

## Introduction

Granulomatous mastitis is a rare benign disease with a prevalence of 2.4 per 100,000 women aged 20-40 years (mean age: 34 years) [1], often occurring within five years postpartum [2,3]. The clinical course of granulomatous mastitis can be self-limiting [4]; however, most patients exhibit a slow resolution of symptoms that can extend beyond one year, and experience many recurrences, thus requiring immunosuppressive therapy to achieve remission [5]. Moreover, in rare cases, granulomatous mastitis can coexist with erythema nodosum [3]. Erythema nodosum refers to septal panniculitis in the extremities and is especially frequently seen in the lower legs. It often occurs in patients with *Streptococcus* infections, tuberculosis, sarcoidosis, and connective diseases such as systemic lupus erythematosus [6].

Erythema nodosum has been reported to occur in postpartum women with granulomatous mastitis. While the associated pathogenesis remains unknown, both autoimmune and infectious causes have been proposed. Hyperprolactinemia has also been reported to cause granulomatous mastitis in women [7]. Granulomatous mastitis with erythema nodosum (GMEN) is often treated with prednisolone [3] due to repeated recurrence and failure to improve with drainage. However, no studies have reported a case of GMEN during pregnancy that has been cured without steroid or immunosuppressant treatment. Here, we report such a case treated via drainage only.

## Case presentation

A 30-year-old G3P1 woman was referred to the Department of Gynecology at 31 weeks of gestation. She noticed swelling and pain in her right breast beginning one week prior to visiting the clinic, where she was diagnosed with right purulent mastitis and received ceftriaxone for four days. However, her symptoms did not improve. As her C-reactive protein (CRP) level and white blood cell (WBC) count remained elevated, she was referred to our hospital for continued treatment. She had no medical or family history of autoimmune disease and had not taken contraceptive pills or antipsychotic drugs.

**Figure 1:**
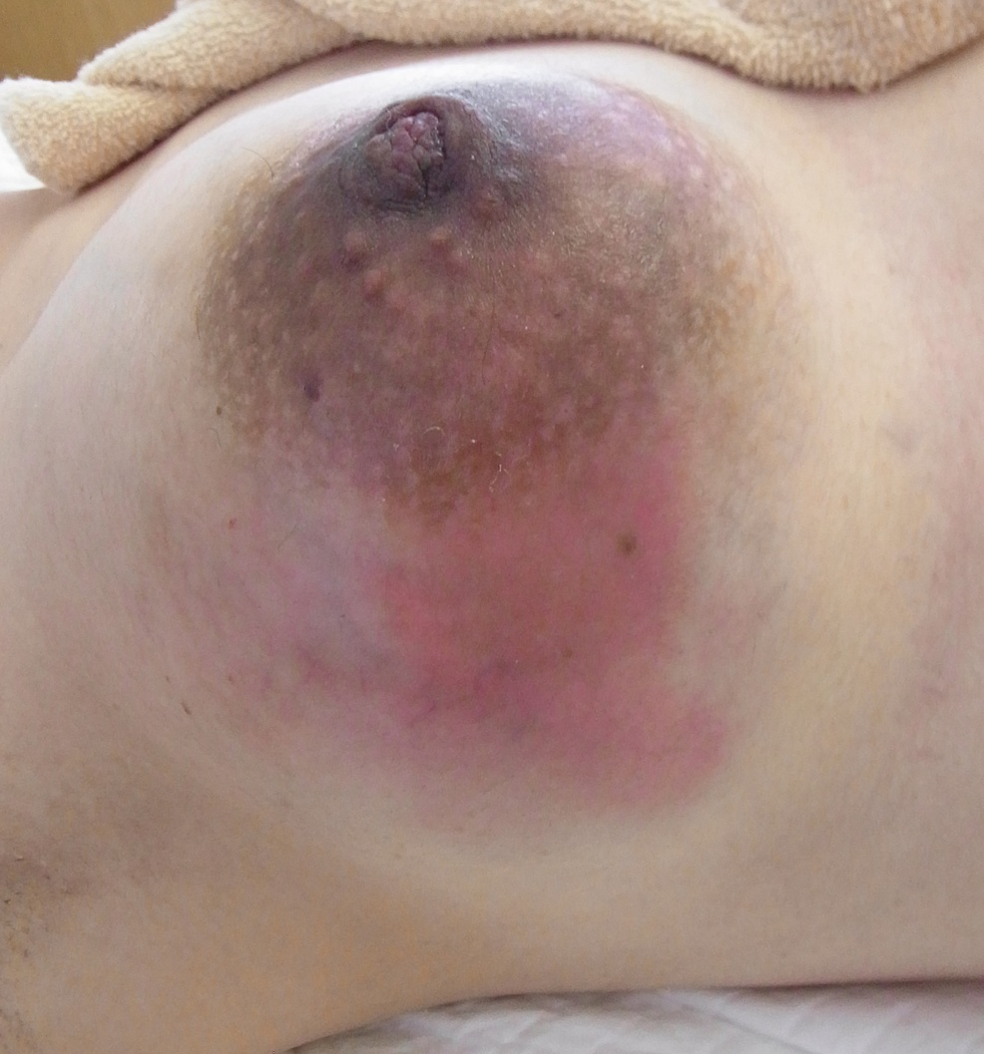
Right breast

**Figure 2:**
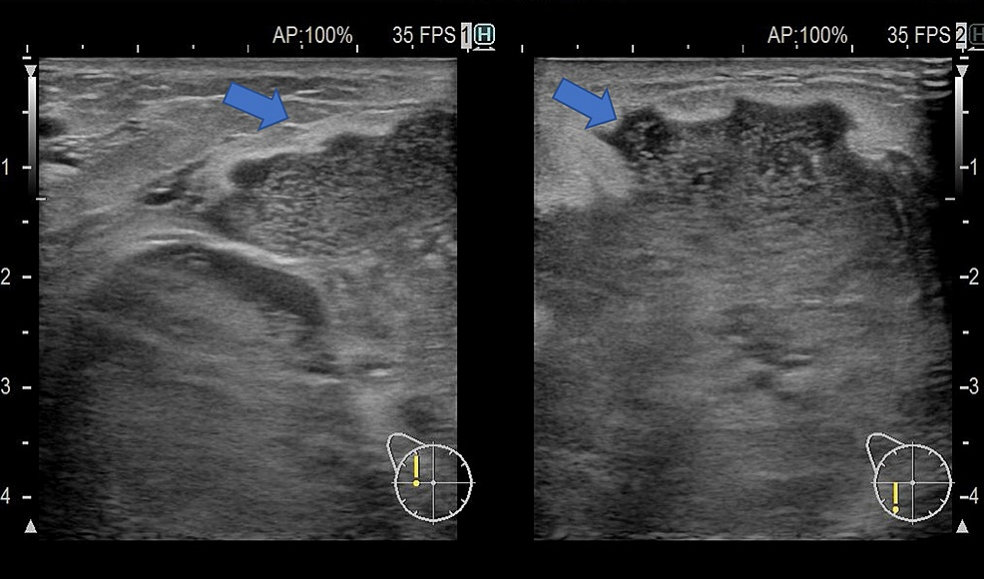
Ultrasonography of the right breast

**Figure 3:**
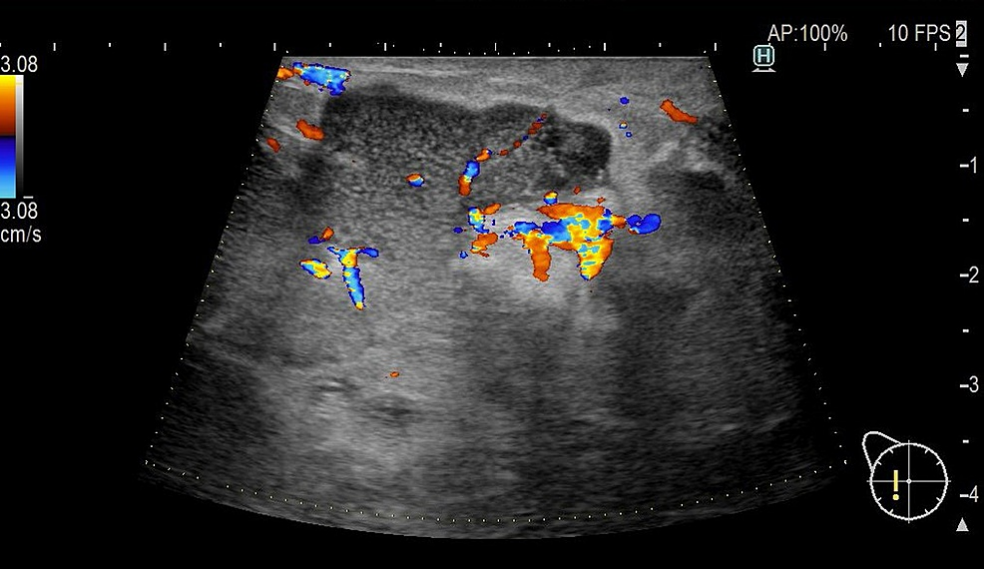
Color Doppler of the right breast

On admission, her body temperature was 36°C. On physical examination, her right breast appeared to be swollen, with a slight nipple discharge (Figure 1). In addition to a nodular mass in the outer quadrants of the right breast with regional axillary lymphadenopathy, a smaller firm mass was detected in the lower quadrants of the left breast. Blood tests revealed a WBC count of 18,150/μL and a CRP level of 4.7 mg/dL (Table 1). Ultrasonography revealed multiple low echoic lesions in the right breast (Figures 2, 3). There was no hilar adenopathy on chest radiography. The abscess in her right breast was drained for diagnosis, and cefazolin was administered. However, her symptoms did not improve. Her body temperature, WBC count, and CRP values were 37.6°C, 13,900/μL, and 2.4 mg/dL, respectively. Because ceftriaxone was administered at a prior clinic, no microorganisms were detected in the pus from her right breast. Thus, we considered mastitis due to antimicrobial-resistant bacteria, breast cancer, or malignant lymphoma.

**Table 1:** Laboratory data on admission Abbreviations: AST, aspartate aminotransferase; ALT, alanine aminotransferase; ALP, alkaline phosphatase; LDH, lactate dehydrogenase; CK, creatine kinase; CRP, C-reactive protein; ASO, anti-streptolysin O; ASK, anti-streptokinase antibody; ACE, angiotensin-converting enzyme; PRL, prolactin; siL-2R, serum soluble interleukin 2 receptor

Marker	Level
Blood cell count	
White blood cells	18,150/μL
Neutrophils	15,972/μL
Lymphoid cells	6.50%
Monocytes	4.00%
Eosinophils	0.50%
Red blood cells	372×10^4^/μL
Hemoglobin	11.2 g/dL
Platelets	50.8×10^4^/μL
Blood chemistry	
Total protein	6.2 g/dL
Albumin	2.5 g/dL
AST	13 U/L
ALT	13 U/L
ALP	169 U/L
LDH	155 U/L
Urea nitrogen	3.3 mg/dL
Creatinine	0.39 mg/dL
CK	11 U/L
Sodium	134.9 mmol/L
Potassium	4.0 mmol/L
Chloride	101.8 mmol/L
Glucose	81 mg/dL
Serological testing	
CRP	4.68 mg/dL
ASO	29 IU/mL
ASK	80
ACE	10.3 IU/L
PRL	196.27 ng/mL
sIL-2R	770 U/L

**Figure 4:**
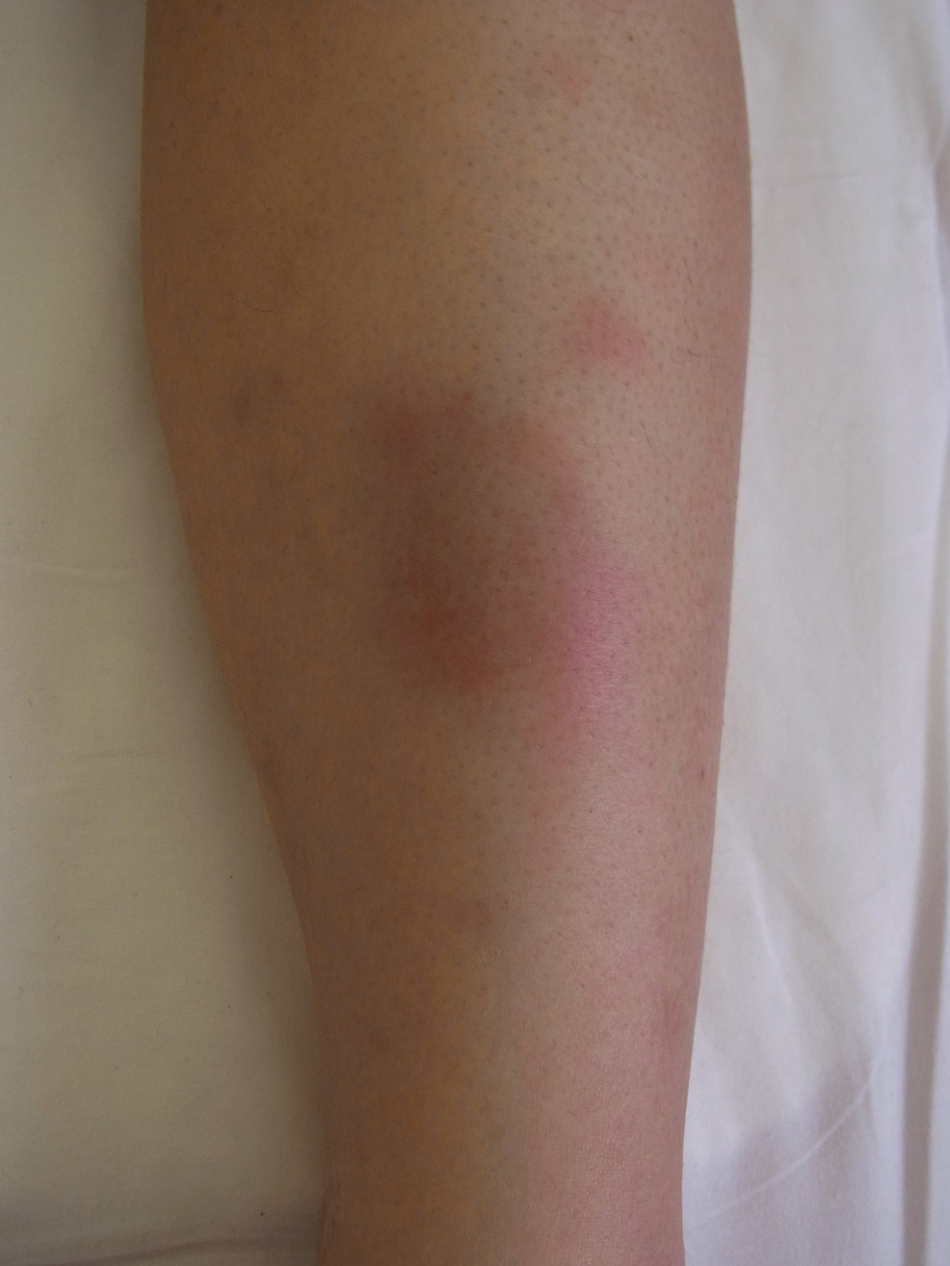
Erythema nodosum in the left lower leg

**Figure 5:**
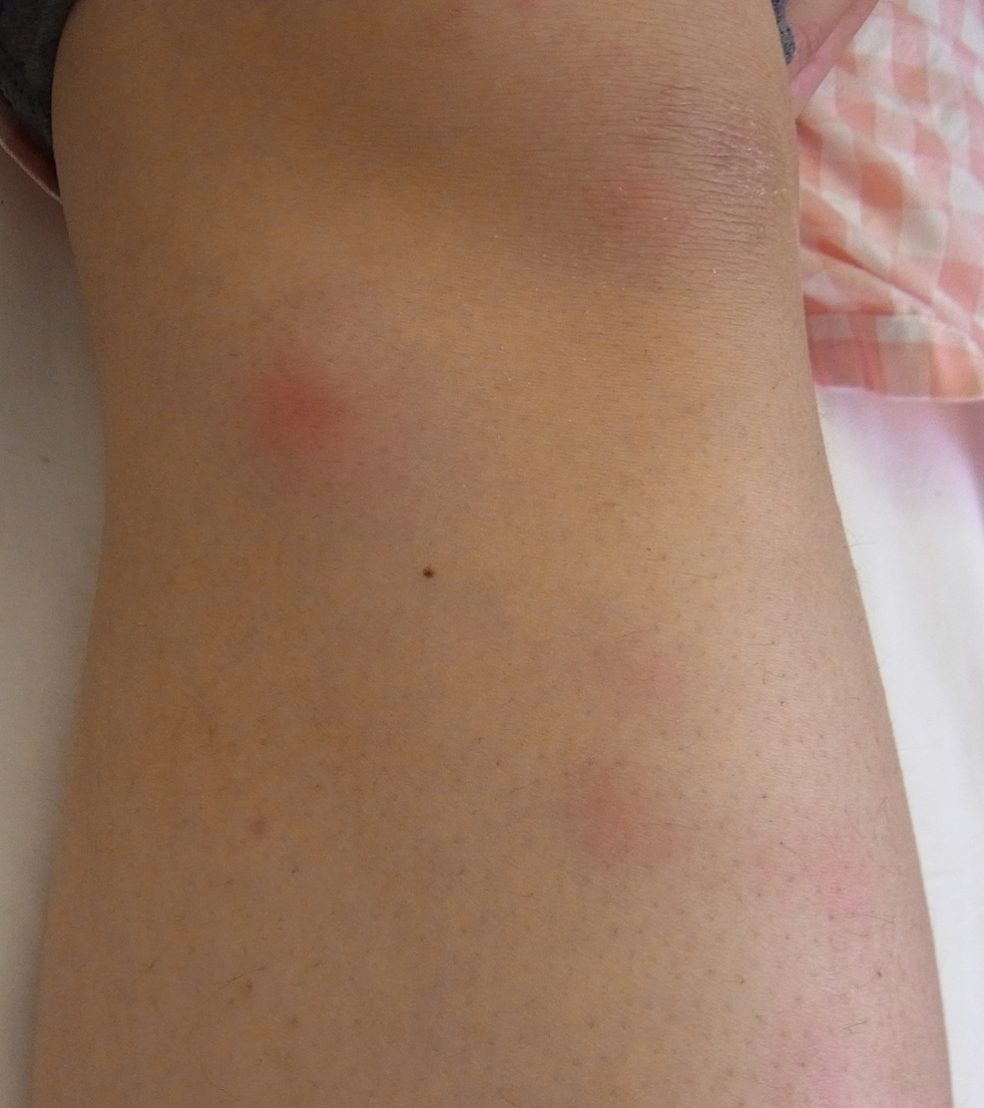
Erythema nodosum in the left lower leg

**Figure 6:**
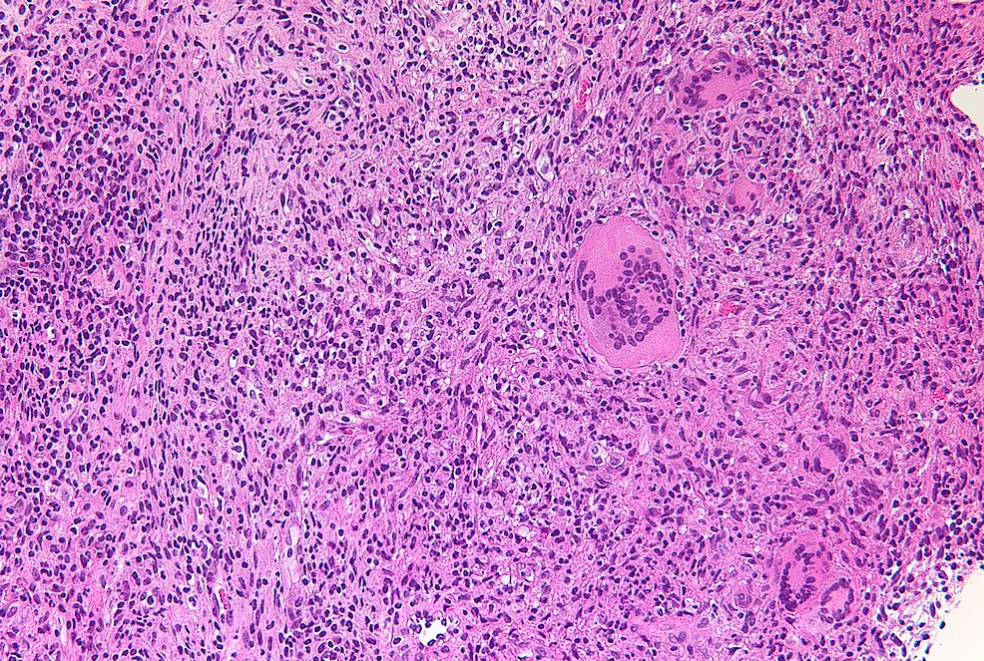
Non-caseating granuloma with epithelioid cells, neutrophils, and lymphocytes (hematoxylin and eosin staining)

Five days after administration, she presented with erythema nodosum in the left lower leg (Figures 4, 5). A fine needle biopsy of the right breast was performed due to suspicion of GMEN. Microscopic examination revealed a non-caseating granuloma with epithelioid cells, neutrophils, and lymphocytes (Figure 6). Acid-fast staining indicated no signs of tuberculosis. Pathologic examination revealed evidence of granulomatous mastitis. After draining the subcutaneous abscess of the right breast several times, the erythema nodosum spontaneously disappeared. Her breast symptoms did not worsen, and she was discharged. She is currently undergoing regular pregnancy examination without any additional treatment.

## Discussion

This case suggests two critical points. First, clinicians should consider granulomatous mastitis when a pregnant woman reports painful swelling accompanied by inflammation in the breast that does not exhibit clinical improvement with antibiotic treatment. Second, pregnant women with GMEN may not need prednisolone treatment due to the anti-inflammatory state associated with pregnancy.

Mastitis generally occurs in postpartum women [2,8], and reports of cases during pregnancy are rare. This is because the mammary glands are closed during pregnancy. Some authors have suggested that granulomatous mastitis is caused by *Corynebacterium kroppenstedtii* [9,10], which is a normal inhabitant of the human skin. Li et al. [11] reported that all 15 pregnant women with granulomatous mastitis in their study had at least one episode before giving birth, indicating that *C. kroppenstedtii* infection may occur during the postpartum period when the mammary glands open. Our patient had one such episode before delivery and did not improve with ceftriaxone or cefazolin treatment, which may be because *C. kroppenstedtii* is generally resistant to β-lactams antibiotics [12]. During the current episode, her symptoms improved without antibiotic treatment, suggesting that some cases of granulomatous mastitis occur in the absence of *C. kroppenstedtii* infection. Given that C. kroppenstedtii is an inhabitant of the skin, related infections can be controlled by the innate immune system after simple drainage to decrease the number of bacteria.

Erythema nodosum is also caused by various triggers, such as group A β-hemolytic streptococcus, sarcoidosis, or pregnancy [3]. In almost all reported cases of GMEN, erythema nodosum appears after granulomatous mastitis, suggesting that it is likely to be a secondary reaction of granulomatous mastitis. Although granulomatous mastitis is rare in pregnant women, it may be associated with prior parity [13,14], especially when symptoms fail to improve with antibiotics and erythema nodosum develops after mastitis.

Many patients with GMEN are treated with prednisolone, with some reports suggesting that long-term treatment is required due to the risk of recurrence. However, in our patient, erythema nodosum gradually resolved, and breast symptoms did not worsen after drainage. Pregnant women have more cortisol in the plasma than non-pregnant women [15]. A high level of cortisol may have allowed for remission of GMEN via drainage only. Indeed, pregnancy has been known to alleviate symptoms in patients with other immunological diseases, such as rheumatoid arthritis. This may help to prevent steroid-associated conditions such as infections and gestational diabetes mellitus.

## Conclusions

Granulomatous mastitis should be considered in pregnant women with treatment-resistant mastitis, especially in those with erythema nodosum and a history of birth. Patients with granulomatous mastitis may not require prednisolone treatment during pregnancy.
